# Highlights from the Special Issue Titled “Recent Advances in Organic Chemistry: Molecules Synthesis and Reactions”

**DOI:** 10.3390/ijms26062787

**Published:** 2025-03-19

**Authors:** Rachid Chahboun, José Justicia

**Affiliations:** Departamento de Química Orgánica, Facultad de Ciencias, Universidad de Granada, 18071 Granada, Spain

## 1. Introduction and Scope of the Special Issue

Organic synthesis, the art and science of constructing complex organic compounds from simpler ones, is one of the most important fields of research in organic chemistry. In fact, numerous Nobel Prizes have been awarded in this field, from the one obtained by Hermann Emil Fischer in 1901 to the latest awards given to authors such as List and McMillan in 2021 or Bertozzi, Sharpless, and Meldal in 2022, confirming the high importance and relevance of this area [1].

This discipline has also impacted other sciences, resulting in the emergence of related fields, such as medicinal chemistry, biology, biotechnology, materials science, and nanotechnology, which depend on advances in organic synthesis to obtain examples of suitable molecules in their studies. Thus, without improvements in organic synthesis, many advances in related disciplines would not be possible.

The development of new synthetic tools and their application to the preparation of various molecules are topics of scientific investigation. Thus, a great variety of synthetic protocols have been developed, including “classic” reactions, organometallic-based reactions, free radical-based reactions, organocatalytic methodologies, photoredox and photocatalysis, and others, expanding the number of synthetic tools available for organic chemists worldwide. These methodologies have allowed for the preparation of an abundance of compounds in laboratories and industries, such as bioactive natural products, nutritional goods, high-tech materials, electronic devices, polymers, and others that are present and facilitate our lives. These interesting objectives increase the relevance of organic synthesis and its advances.

In this Special Issue, we have compiled an interesting collection of 10 contributions in different fields of organic synthesis, significantly contributing to the advancement of this discipline, providing new synthetic tools for the achievement of complex synthetic objectives, and for the preparation of molecules of interest, such as bioactive and photosensitive compounds. This editorial summarizes the contributions to this Special Issue, describing the published research, applications, and potential future directions, thereby providing a comprehensive overview of the contents of this collection.

## 2. Highlights of the Special Issue

### 2.1. Synthesis and Biological Activity Evaluations

One of the main applications of organic chemistry is the preparation of bioactive compounds using short and efficient procedures. This constitutes an important goal in this field [2], and great efforts have been made to develop complex structures and highly bioactive drugs. This Special Issue includes four contributions in this area. Rosamines are a promising compound with a xanthene structure, with excellent photophysical properties and applications [3], especially as photosensitizers in the field of photodynamic therapy. Mernyák et al. describe a novel procedure for synthesizing several rosamine compounds from pyridylbenzaldehyde derivatives using an effective microwave-assisted process. The photophysical properties of the synthesized pyridyl-rosamines were studied, mainly using UV-Vis absorption and fluorescence emission spectra, showing interesting results. In addition, the authors examined the cytotoxic effects of these compounds against the A431 human epidermoid carcinoma cell line to determine their potential as photosensitizers. The results indicated that these compounds could display light-induced toxicity in the submicromolar or low nanomolar range. The authors particularly emphasized the activity of compound **16**. These results indicate that these compounds are promising phototheranostic candidates suitable for combined fluorescence diagnosis and photodynamic therapy (PDT).

Gao, Li et al. contributed to this Special Issue with a remarkable article on the synthesis of 3,4-disubstituted derivatives of maleimide compounds. Maleimides are moieties of pyrrole-2,5-diones frequently found in the structure of natural products and bioactive compounds [4], such as the antibiotic rebeccamycin [5] or the indolylmaleimide derivative, an inhibitor for cancer cells [6]. However, the synthesis of such interesting molecular structures remains a challenge for organic chemists. To solve this problem, the authors developed a new approximation for the synthesis of 3,4-disubstituted maleimide derivatives based on the direct isomerization of α-succinimide-substituted allenoates, followed by a cascade γ′-addition, and aryl imines using phosphines as a catalyst. Using a model compound, the authors optimized the reaction conditions and efficiently synthesized 18 derivatives in moderate-to-good yields. The authors also proposed a possible mechanism for this reaction. This work offers a good methodology for accessing these interesting compounds.

The synthesis of new compounds with antitumor activity is a relevant objective in organic synthesis [7,8]. In this context, Pan et al. report the design and preparation of 21 furanopyridone derivatives from 3-furan-carboxylic acid. In a few steps, the authors converted the starting acid into the derivatives **2d**–**2f**. Furthermore, from intermediate **2b**, they prepared additional derivatives **3a**–**3k**, by the alkylation of the N atom of pyridone. One of the compounds obtained, compound **3e** was used in the synthesis of the furan [3,2-c] pyridine derivatives **4a**–**4d** by the alkylation of the corresponding bromo-derivative. Altogether, the authors prepared a significant number of products with great structural diversity. Subsequently, they studied the antitumor activity of these compounds against the KYSE70 and KYSE150 cell lines. In general, most of the compounds studied exhibited significant antitumor activity. In particular, compound **4c** was shown to be the most active, with an inhibition percentage of 99% in both cell lines. To better understand the behavior of this compound, the authors performed molecular docking studies of **4c** against the EGFR (PDB ID: 6DUK) and MetAP2 (PDBID: 5D6E) proteins. This study exemplifies the importance of organic synthesis in the development of new therapeutic tools.

Other important drugs for treating cancer diseases include microtubule-targeting agents, which have a high effectiveness against cancer but have limited clinical applications because of several drawbacks [9]. Alternatives to introduce these important tools into common therapeutic treatments for cancer are desirable. Tobiasz, Krawczyk et al. present the synthesis of biphenylmethoxydibenzo [b,f] oxepine or photoswitchable fluorinated dibenzo [b,f] oxepine derivatives, with a potential activity as microtubule inhibitors. The authors efficiently prepared several derivatives (**2a**–**2j**, **6a**, **7a**–**7e**, and **9a**–**9f**) with complex dibenzooxepine structures. Then, they explored the switchable properties of the obtained compounds. It is noteworthy that compound **9e** provides a basis for further improvement and development of novel photoswitchable dibenzo [b,f] oxepine-based microtubule polymerization inhibitors.

### 2.2. New Synthetic Processes

The development of new synthetic tools, in fact, new chemical reactions and/or new applications for previously described processes, constitutes an important and attractive goal in the field of organic chemistry [10]. Three interesting contributions to this research topic are included in this Special Issue. C (sp^2^)-Cl fragments are present in a great number of molecules, such as natural products, pharmaceuticals, agricultural chemicals, and other synthetic intermediates [11]. Several methodologies for the direct chlorination of C (sp^2^)-H bonds have been described, using various reagents (Cl_2_, SOCl_2_, NCS, etc.) [12]. The described methodologies typically use solvents that can introduce some disadvantages. Some examples using 1,2-dichloroethane as a solvent and Cl atoms source have been described [13]. However, these methodologies are focused on the halogenation of aryl C (sp^2^)-H bonds, and no examples of the chlorination of vinyl C (sp^2^)-H bonds are described. To address this issue, Wang et al. developed a new synthetic methodology for the chlorination of vinyl C(sp^2^)-H using 1,2-dichloroethane as the solvent and Cl atom source, following a metal-free methodology. This reaction was applied to enaminones, producing the corresponding α-chlorinated derivatives with *Z*-configuration in good-to-excellent yields. In this work, the authors provide organic chemists with a new tool for the chlorination of vinyl C (sp^2^)-H bonds and, specifically, enaminone derivatives.

Another interesting field of study in organic synthesis is the development of efficient procedures for nucleophilic aromatic substitution reactions, which can be used in the preparation of interesting molecules, such as building blocks for functional materials and heterocycles [14]. In this regard, some halogenated aromatic compounds are excellent substrates for these processes. Thus, Plater et al. contributed to this Special Issue by synthesizing heterocycles such as dioxins, dithiins, and phenoxathiines using an efficient nucleophilic substitution of activated fluorinated aromatic compounds under mild and basic conditions. The structures of the synthesized heterocycles were determined by single-crystal X-ray single crystallography. Additionally, other heterocycles were prepared from commercial 2,3-dichloroquinoxaline using the developed methodology. The obtained compounds were used to synthesize sulfone derivatives such as **19** and other *N*-alkyl derivatives.

One important aspect of modern organic synthesis is the development of new processes using fewer contaminants and without environmental disadvantages. Usually, solvents used in organic synthesis are environmentally problematic because of their toxicity and the high amount of volume required in any organic reaction. Thus, new reactions using environmentally friendly solvents and reagents are an important goal. To reach this objective, Mándity et al. presented an example of a reaction performed using a green and eco-friendly solvent, propylene carbonate (PC) [15]. They used this solvent in the *N*-alkylation of N-, O- and S-containing heterocycles with a 2-hydroxypropyl moiety, and indicated that PC acts as a solvent and an alkylating agent in the reaction. All reactions were efficient, yielding the corresponding monoalkylated compounds. In the case of 6-methyluracil (**5**) and 2-thiouracil (**7**), dialkylated compounds were obtained. Additionally, the authors claimed that the reaction was also efficient in the case of benzotriazole (**6**). The experimental data obtained in these experiments were supported by theoretical calculations.

### 2.3. Structural Studies

The determination of complex 3D structures in organic molecules constitutes a prominent field of study. Their biological and technological applications have promoted the preparation of these compounds using organic synthesis protocols. Nevertheless, the development of alternative and synthetic tools is desirable. In this Special Issue, there are two contributions to this field. Plater et al. also contributed to this Special Issue by studying single-crystal X-ray structures of a series of compounds with quinoxaline and pyrimidine structures. These heterocyclic compounds were prepared using the described nucleophilic aromatic substitution reaction. The structural studies indicated that all compounds were densely packed without porosity. The authors also determined that compounds **16** and **18** present the same hydrogen bond pincer motifs, which conditions the final structure of these compounds. Interesting conclusions were also obtained regarding the structures of compounds **12**, **14**, **19**, and **21**, providing important information for chemists interested in this field.

Sulfoxides and sulfinate esters have been recognized as optically active compounds for decades [16]. Chiral sulfoxides are important in the synthesis of asymmetric organic compounds and drugs. Related to these compounds are racemic thianthrene sulfoxides, a rare family of compounds with some remarkable examples, such as 1-methylthianthrene-5-oxide, 2,7-dimethylthianthrene-5-oxide, and chlorpromazine-S-oxide. However, enantiomerically pure or enriched compounds of this type are more useful. In their contribution, Plater et al. described the synthesis of enantiopure sulfoxides from the model thianthrene **3**. The authors used the nucleophilic aromatic substitution reaction described above to synthesize the thianthrene skeleton in **6**. Subsequently, the monoxidation of sulfur atom, followed by the displacement of the NO_2_ group with (S)-phenylethylamine, was conducted to the corresponding enantiomerically pure thianthrenes **9** and **10**, which are diastereomers. These compounds were characterized by single-crystal X-ray diffraction analysis.

### 2.4. Applications for Materials Chemistry

Another interesting goal of organic synthesis is the construction of complex molecular platforms that can be used to obtain interesting organic materials, such as polymers, cyclodextrins, molecular devices, and fullerenes. The development of efficient and accessible methods for the construction of complex structures has become an important field in organic synthesis. One example is “click chemistry”, which was introduced early by Sharpless et al. [17] and has several applications [18]. One of these applications is the preparation of complex materials for nanomedicine. In this Special Issue, Guadarrama et al. provided a review of this topic. They described the main contributions of “click chemistry” in the synthesis of polymers, cyclodextrins, and fullerenes, with special attention on the functionalization of these compounds with bioactive molecules (drugs or biomolecules) using several “click chemistry” procedures, such as copper-catalyzed azide-alkyne cycloaddition, strain-promoted azide–alkyne cycloaddition, inverse electron-demand Diels–Alder, and thiol–ene click reactions. The application of these reactions has allowed the preparation of sophisticated systems with improved properties, which are essential in the field of nanomedicine.

## 3. Conclusions

This Special Issue showcases the significant impact of organic synthesis in science. The research articles and review published in this Special Issue represent interesting examples of the various efforts being made to identify new chemical processes with applications of interest, with special relevance to the field of bioactive products. These results may serve as an inspiration for other authors to continue their studies in this field and contribute to the development of organic synthesis worldwide.
